# The Role of Personality in Prediction of Satisfaction With Life in Recreational Athletes During the First Wave of Pandemic Covid-19

**DOI:** 10.3389/fpsyg.2021.820045

**Published:** 2022-01-20

**Authors:** Danijela Živković, Jasmina Nedeljković, Bojan Veljković, Anđela Đošić, Željka Bojanić, Milovan Bratić, Saša Pantelić

**Affiliations:** ^1^Faculty of Sport and Physical Education, University of Niš, Niš, Serbia; ^2^Faculty of Law and Business Studies Dr Lazar Vrkatić, University Union Belgrade, Novi Sad, Serbia; ^3^Academy of Educational and Medical Vocational Studies, Kruševac-Cuprija Department, Subotica, Serbia

**Keywords:** personality, satisfaction with life, individual recreational sports, pandemic Covid-19, university students

## Abstract

The aim of this research is to contribute to the understanding of the concept of satisfaction with life by determining the relationship between personality traits and the subjective experience of satisfaction with life in students—recreational athletes. This research is based on the biological theory of personality by Hans Eysenck and it attempts to offer explanations of a possible change in satisfaction with life in the period of great social deprivation caused by the Covid-19 pandemic. The sample of subjects consisted of 120 undergraduate students (*N* = 120) of all years and both sexes, 55 (45.8%) males and 65 (54.2%) females, at the Faculty of Sport and Physical Education, the University of Nis. The average age of the subjects was 23.63 years (SD = 2.070). Eysenck's personality questionnaire (Eysenck Personality Questionnaire, EPQ: Eysenck et al., 1885, adapted and translated by Šipka, 1985) was used for the operationalization of personality structure. The SWLS scale (Satisfaction With Life Scale, Diener et al., 1985) was used for estimating satisfaction with life. A significant regression model, which explains 11% of variance in the subjective experience of satisfaction with life in recreational athletes, was obtained. In the model, extraversion stands out as a significant predictor from the group of personality traits (β = 0.279). Neuroticism (β = −0.160) and psychoticism (β = −0.122) did not prove to be significant predictors of satisfaction with life in the structural model regardless of there being a significant negative correlation between neuroticism and satisfaction with life. The more extraverted participants had a keener subjective sense of satisfaction with life.

## Introduction

According to a popular metaphor, the processing of information in the human brain is analogous to the processing of information inside a computer. Our mental states can, therefore, be considered as “algorithms” of information which vary in respect of entrance and exit, whereas there is no essential difference in the processing itself (Bender and Beller, 2011). However, it is an undeniable fact that there are enormous differences in how and what we perceive, how we think about the perceived, how we feel afterwards, whether or not we are basically satisfied. Our general satisfaction, as well as the satisfaction with our own lives, is a combination of cognitive, affective and behavioral dimensions. Different personality features/dimensions determine to what extent we will be psychologically adapted to the world we live in and also how satisfied with our own life we will feel. Today's digital society offers young people numerous possibilities, but at the same time, brings frequently implicit risks, which can have consequences on personal well-being and also affect the self-evaluation of one's own satisfaction with life (Mascia et al., 2020). Certain studies have confirmed that personality traits in young people significantly contribute to the explanation of the variance of one's satisfaction with life (Tuce and Fako, 2014). It has been determined by meta-analysis of numerous studies of the relationship between personality traits and satisfaction with life that the contribution of personality traits in the explanation of satisfaction with life varies from 39 to 63% (DeNeve and Cooper, 1998). It is known that satisfaction with life is not only influenced by numerous stable personality traits but also by social environment factors. A new “variable,” which has been present in our lives since 2019, the Covid-19 pandemic, has led to a number of consequences in context of mental health of the general population. As its common denominator, it includes anxiety, depression, problems with sleeping, diseases of the muscle-bone tissue due to reduced movement, abuse of alcohol and other psychoactive substances, etc. In reference to the self-protective behavior, there have been a large number of various emotional and behavioral responses to the new situation (Lep et al., 2020). Basically, it was often the case of the need to avoid loneliness because some studies have shown that a higher degree of loneliness was connected to reduced satisfaction with life (Begčević, 2021). An enhanced need for movement, going out for a walk, recreational running, going to gyms and fitness centers, was a common behavioral response to the crisis in a certain group of young people with an interest in sport. However, even before the pandemic, doing sport was not a full guarantee that mental health would remain safe from episodes of anxiety and depression. Meta-analysis which examines anxiety among athletes has pointed to an association in the 75% of the included research (Rice et al., 2016). It is a widespread opinion that athletes and “non-athletes” differ in many personality traits (Geron et al., 1986). A large number of studies have clearly shown that athletes are more independent, more objective and less anxious than those people who do not do any sport. This was confirmed by Morgan (1980) who reached a conclusion that athletes are basically extraverted and with low anxiety. With regard to determinants of satisfaction with life during the Covid-19 pandemic, we had in mind a large number of research findings which have confirmed that personal reactions to life events take predominance over the events themselves and that personality traits impact the kind of reactions one will have (Diener and Diener, 1996; Schimmack et al., 2004). Involvement in pleasant activities during the pandemic could have had an indirect effect on the feeling of satisfaction with life both on the affective and cognitive level (Gutiérrez-Cobo et al., 2021). As Pavot and Diener (2013) point out in their study, despite the many research findings, a relationship between personality traits and satisfaction with life, significant and frequently consistent as it may be, has still not been completely investigated. Based on Eysenck's theory of personality, and the social deprivation caused by the Covid-19 pandemic, we hypothesized that the structure of the subjective self-evaluation of satisfaction with life can be explained by extraversion-introversion, neuroticism, and psychoticism personality traits in recreational athletes. Since these premises indicate the need to investigate the factors which might affect the feeling of satisfaction with one's life in recreational athletes, this study will investigate multivariate relationships, the connection as well as the effects of personality traits on self-evaluation of satisfaction with one's life within the specified part of the population. This research was conducted during the Covid-19 pandemic while various epidemiological measures were in power.

### Personality Traits

Among other things, in Eysenck's theory of personality (Hall and Lindzey, 1978), the importance of external, manifesting forms of behavior for the appropriate understanding of personality has been emphasized. We explain the habit of going to individual training sessions to fitness centers or gyms with the second level in the structure of personality which Eysenck named “common reactions, habits or actions inherent in one personality.” The third level of organization of personality represents features or traits of personality, while the fourth and the highest level of organization of personality, Eysenck names *types of personality*. This descriptive aspect of Eysenck's biologically founded model of personality refers to hierarchical taxonomy obtained through factor analysis in which three dimensions of personality were extracted: introversion–extraversion (IE), neuroticism (N), and psychoticism (P). Eysenck names these dimensions types of personality (Hall and Lindzey, 1978). Eysenck described dimensions of personality considering their causal aspect. Thus, he determined that extraversion is based on cortical arousal which can be measured with skin irritation, brain waves or sweating. By theoretical assumption, there is an optimal level of arousal over or under which the performance gets worse. Extraversion is connected with interest in companionship and positive feelings. Diener and Larsen (1993) did not confirm the assumption that extroverts have an increased interest in companionship and positive feelings based on the length of time they spend with other people. One of the possible explanations is that dopamine effects the interest in companionship and positive feelings, making people highly sensitive to rewards. Neuroticism is based on activation threshold in sympathetic nervous system or visceral nervous system. This is the part of the brain that is responsible for the “hit or run” response while facing danger. Activation can be measured with the heart rate, blood pressure, cold hands, sweating, and muscle tension (especially in the forehead area). Neurotic people, who have a low activation threshold, experience a negative feeling (hit or run) even in the least stressful situations, they are easily triggered (Servaas et al., 2013). Emotionally stable people with a high activation threshold, experience a negative feeling only in highly stressful situations, they keep their heads even under pressure. It is interesting to point out that physiological indicators of neuroticism do not correlate highly between each other. Namely, people react differently in stressful situations. Some sweat, others get a headache. This is called an individual specificity of response. It is also interesting that different stressors provoke a different response. This is called stimulus specificity of response. Psychoticism is connected with a tendency for psychotic episodes (or disconnecting with reality) and aggression (Nederlof et al., 2011). Although less research was done on psychoticism than on extraversion and neuroticism, researchers are completely assured that the basis of psychoticism is biological and that it is caused by increased levels of testosterone (Tajima-Pozo et al., 2015; Lodha and Karia, 2019).

### Satisfaction With Life

Satisfaction with life is a construct from a domain of positive psychology. It is defined as a cognitive component of subjective well-being (Diener et al., 1999). Satisfaction with life is commonly used as a synonym for happiness (Diener and Ryan, 2009). The main focus in a large number of studies of satisfaction with life was in correlation with other psychological and social variables. A series of studies in literature indicate that many factors affect self-assessment of the quality of life in young people (Jovanović, 2016). In attempts to theoretically explain the concept of satisfaction with life, various models were developed. These models can be sorted in two categories considering the fact whether they emphasize an ascending or a descending perspective in studying the factors that have an effect on satisfaction with life (Diener and Ryan, 2009). Thus, theories based on the descending perspective, the so-called top-down models, rest on the assumption that the satisfaction with life is something that is relatively stable. In other words, there is a general tendency to experience different situations positively or negatively. This approach is commonly directed at personality traits and different cognitive evaluations as factors that affect satisfaction with life (Diener and Diener, 1996; Diener and Ryan, 2009). In contrast to this, theories based on the ascending perspective, the so-called bottom-up models, rest on the assumption that the global feeling of satisfaction forms on the basis of adding satisfactions from different domains of life, such as marriage, job and family. In contrast to the descending perspective, objective circumstances have a more important role here, and the satisfaction with life is generally seen as something relatively unstable, depending on shifts in positive and negative experiences (Diener and Diener, 1996; Diener and Ryan, 2009). The contemporary perspective of the determinants of satisfaction with life rejects an exclusive focus on either the descending or ascending perspective and points to a necessity for a kind of synthesis and integration of the previous two models. In this context, Dynamic Equilibrium Model or, as the author calls it in more recent times, Set-Point Theory (Headey, 2006a,b, 2008), represents a dominant contemporary model of the determinants of satisfaction with life. It includes both the effect of different dimensions of personality on an evaluation of satisfaction with life and specific objective indicators and subjective changes, as factors that affect satisfaction with life (Headey, 2008). Therefore, according to the contemporary understanding of determinants of satisfaction with life, dimensions of personality should certainly be viewed as the most important predictors of satisfaction with life, not disregarding the impact of different life experiences which should be considered as well (Diener, 2013; Headey et al., 2013).

### Personality Traits and Satisfaction With Life in Recreational Athletes

Numerous studies have confirmed a connection between doing a physical activity and satisfaction with life as one of the components of subjective well-being. Diener et al. (1999) recognized a dramatic increase in the interest in determinants of subjective well-being as well as in scientific research of these determinants. The research shows a proven distinction between the affective and cognitive component of the subjective well-being. Also, one group of scientific papers confirmed that socio-demographic characteristics (age, gender, educational and marital status), health and income have a negligible role in explaining the variance in satisfaction with life (Campbell et al., 1976). Another group of scientific papers showed that satisfaction with life is stable through time and that it is often in correlation with similarly stable personality traits (DeNeve and Cooper, 1998; Diener et al., 2003). A study conducted by Steel et al. (2008) showed that personality traits explain the variance in subjective well-being of up to 39% or 63%, corrected from the error of measure. An integrative study which studied the stability of satisfaction with life (Schimmack et al., 2002) showed that tree personality dimensions—extraversion, neuroticism and conscientiousness—can explain 65% of the variance of satisfaction with life. Some studies showed that satisfaction with life can be viewed as the result of satisfaction with different life domains (Andrews and Withey, 1976; Campbell et al., 1976).

Sport is a very important life domain and can be viewed as a domain of satisfaction with life unrelated to work. In sports literature, the subjective point of view of an athlete is largely neglected either in favor of the objective performance score, the physical and technical qualities or is otherwise based on the concept of achievement and the goal theory (Sit and Lindner, 2005). In one study (Baudin et al., 2011), the main goal was to replicate relationships between dimensions NEO PI-R and satisfaction with life as were established in previous research (DeNeve and Cooper, 1998; Schimmack et al., 2004; Steel et al., 2008) on the sport-oriented sample; the hypothesis that extraversion and neuroticism were significant predictors of satisfaction with life was tested and confirmed here (Diener et al., 2003). In the research of subjective well-being conducted in recent years, emphasis was placed on sport, exercise and physical activity because previous studies consistently showed their connection with satisfaction with life (e.g., Huang and Humphreys, 2011; Höner and Demetriou, 2012; Richards et al., 2015; Sigvartsen et al., 2016; Dolan et al., 2017). Positive correlations between physical activity and subjective well-being were found in all age groups, including young ones (McMahon et al., 2017), students (Jetzke and Mutz, 2020), adults (Downward and Dawson, 2016; Marques et al., 2016), and the elderly (Lera-Lopez et al., 2019). One of the more significant findings (White et al., 2017; Wiese et al., 2018; Zhang and Chen, 2019) is that even small amounts of additional physical activity lead to an increase in well-being. Recreational sports activities and spending more hours on these activities are related to higher well-being (Oishi et al., 2007; Diener and Ryan, 2009; Vinson and Ericson, 2014). Practicing sport voluntarily may also contribute to the well-being and its component life satisfaction.

### Research Question

Taking into consideration the fact that Eysenck's theory of personality is biologically based and the social deprivation of all people caused by the Covid-19 pandemic, we have formulated a research question: Can the structure of the subjective self-evaluation of satisfaction with life be explained by extraversion-introversion, neuroticism and psychoticism personality traits in students- recreational athletes?

## Materials and Methods

### Particpitants

One hundred twenty undergraduate students of all years at the Faculty of Sport and Physical Education, the University of Nis (*N* = 120) were surveyed. The sample consisted of 55 (45.8%) males and 65 (54.2%) females of the average age of 23.63 years (SD = 2.070), the mode of age being 23. The surveyed students were non-competitors and did individual sports, such as karate, judo, jiu-jitsu, kick box, taekwondo, power lifting, HIIT, etc. for recreational purposes. On average, they trained these sports for 11.03 years (SD = 3.42). Inclusion criteria were as follows: the subjects were healthy without having Covid-19 infections during the first wale of the pandemic; before the first wale of the pandemic, they were physically active at least three times a week, at least 1 h per session, participating in the recreational activities. The exclusion criterion was participation in competitive sports.

### Instruments

Eysenck's personality questionnaire, which measures dimensions of neuroticism, extraversion and psychoticism (*Eysenck Personality Questionnaire, EPQ: Eysenck et al.*, *1885**, adapted and translated by Šipka*, *1985*) was used for operationalization of personality structure. EPQ consists of 90 dichotomous items arranged in 4 scales: E-extraversion (21 items), N-neuroticism (23 items), P-psychoticism (25 items), and L-scale (21 items). The results of research on the student population (Emić et al., 2015) showed that the reliability of the questionnaire according to Gutman-Cronbach's alpha is: P-scale = 0.61, E-scale = 0.86, and N-scale = 0.85.

SWLS-satisfaction with life scale was used as an instrument for estimating satisfaction with life (Satisfaction With Life Scale, Diener et al., 1985). The scale is made of five claims and a respondent estimates their level of agreement with the stated claims on a seven degree scale of Likert type. Scores can vary in the range of 5–35, where higher scores indicate higher satisfaction with life. SWLS-scale measures the cognitive component of personal well-being.

### Procedure

The research was carried out during May 2020, with previously obtained approvals from the ethical committees of the Faculty of Sport and Physical Education, the University of Nis and voluntary consents of the students. We have provided the anonymity of the collected data. All subjects gave written informed consent in accordance with the Declaration of Helsinki. The testing was conducted in the classroom, in a paper-and-pencil test form and it lasted for 20 min.

### Data Analysis

In the data analysis, descriptive measures, mean differences, and structural equation modeling were used. Statistical processing was done using the statistical packages SPSS and Amos (Arbuckle, 2014. Amos (Version 23.0) [Computer Program]. Chicago: IBM SPSS) (Table 1).

**Table 1:** Means, standard deviations, and correlation between variables.

	**Minimum**	**Maximum**	**Mean**	**Std. deviation**	**Corr. SWLS with…**
Satisfaction with life	14	35	25.65	4.840	–
Extraversion	4	21	14.27	4.268	0.293^**^
Neuroticism	1	21	10.11	4.883	−0.248^**^
Psychoticism	0	13	4.29	2.639	−0.081
Valid N (listwise)					

*Corr. = Pearson's correlation between satisfaction with life and personality traits*;

** *Correlation is significant at the 0.01 level (2-tailed)*.

## Results

Statistically important correlations between satisfaction with life and extraversion (positive) and neuroticism (negative) were established. More extraverted recreational athletes were more satisfied with life than introverts. Also, recreational athletes with low neuroticism were more satisfied with life.

### Research Question

Can the structure of the subjective self-estimation of life satisfaction in students- recreational athletes be explained with personality traits, such as extraversion-introversion, neuroticism and psychoticism?

**Figure 1:**
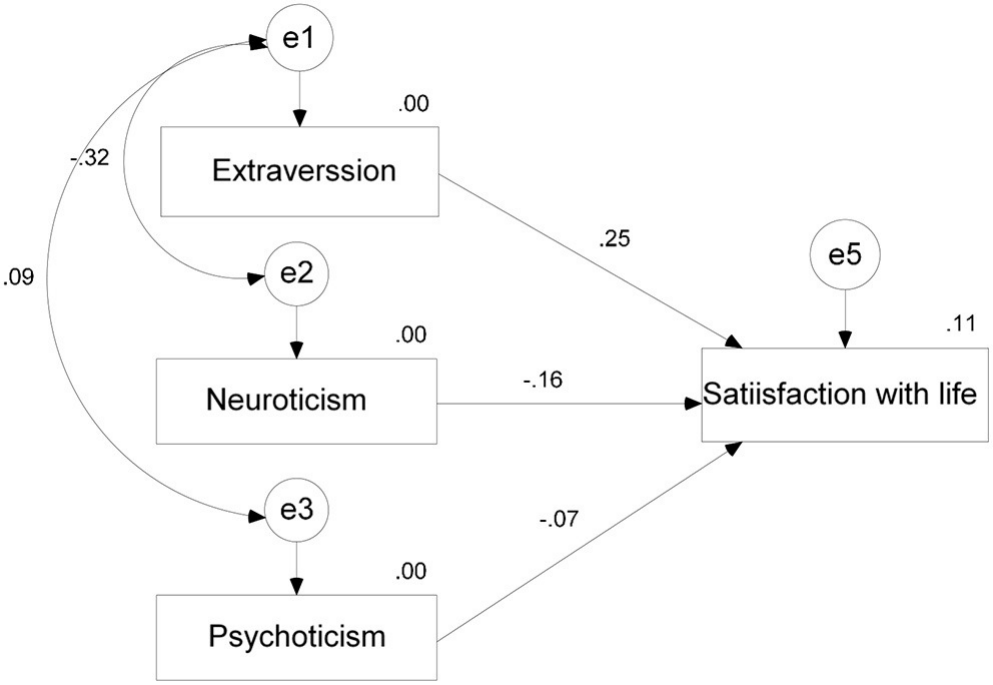
Model–life satisfaction explained with personality traits.

In the modeling process, an assumed direction of the relationship between the XY variables was chosen whereby X represents personality traits as an independent variable and Y represents life satisfaction as a dependent variable. Interrelationships between personality traits were also defined. After defining the model, its testing was conducted in accordance with the hypothesis. Re-specification of the model was conducted through the analysis of statistical significance of regression coefficients and the analysis of the residuals whose high values indicate the existence of correlations which were not included in the model. The model also included relationships which were indicated by the higher residual values between variables. The best model that we established includes three dimensions of personality–neuroticism, extraversion, psychoticism and residual correlations between neuroticism and extraversion and between extraversion and psychoticism. Fitting parameters of this model proved to be best of all models tested. This model explains 11% of the variance in the life satisfaction variable (Figure 1).

From the value of the indicators of the model's goodness of fit which was rated as the best of all done in the analysis given in Table 2, it can be seen that all fit indices exceed critical values (Milas, 2009), and that χ^2^ is statistically significant. However, as the connections that were not statistically significant were excluded in the previous step, it has been estimated that it would be more accurate to keep this model.

**Table 2:** Fitting parameters.

**χ^2^ (6)**	**RMSEA**	**GFI**	**AGFI**	**NFI**	**CFI**
2.833	0.124	0.988	0.884	0.908	0.926

*p = 0.000*.

By analyzing regression coefficients (Table 3), it has been observed that in this combination of relationships between variables, another connection becomes irrelevant (Neuroticism (β = −0.160, *p* = 0.06), but it has been decided not to modify the existing model any further with an assumption that on a larger sample that connection may be of statistical importance, as has been established in a number of previous studies. On the basis of regression coefficients it can be concluded that all correlations are low or are in between low and medium intensity.

**Table 3:** Regression weighs.

			**Estimate**	**S.E**.	**C.R**.	***P***
SWLS	< –	N	−0.160	0.085	−1.875	0.061
SWLS	< –	P	−0.122	0.158	−0.774	0.439
SWLS	< –	E	0.279	0.098	2.853	0.004

## Discussion

A change in life circumstances which has occurred due to the Covid-19 pandemic has particularly affected, apart from physical health, people's functioning in the social segment which is the most significant factor in explaining mental health and well-being. In these altered life circumstances caused by the application of various measures for the prevention of the spread of the virus, all aspects of health, including physiological, psychological and social, were put to the test. These new circumstances on the world level have raised a large number of questions. One of these questions is the topic of interest of this work which discusses whether personality determinants such as extraversion, neuroticism and psychoticism, can overpower the confusion with the information related to the Covid-19 pandemic in young people who do spots recreationally and thus increase their subjective feeling of satisfaction with life. Based on this open question, the basic assumption of this work has been formulated–personality traits can be significant predictors of satisfaction with life regardless of the external circumstances. A negative association between neuroticism and satisfaction with life was established (Bratko and Sabol, 2006), which proves its irrelevance in the model and goes in favor of the researchers' claim about the uncertain role of personality dimensions in satisfaction with life (Sato et al., 2018). Low neuroticism is associated with higher satisfaction with life. Extraversion in our sample is positively associated with satisfaction with life and this finding is in accordance with all former research (Campbell et al., 1976; Emmons and Diener, 1985; Eddington and Shuman, 2004; Schimmack et al., 2004). However, as opposed to neuroticism, the role of extraversion in the tested model of satisfaction with life is also significant. Scientific evidence confirms that leisure time physical activity represents an important correlate with satisfaction with life (Sato et al., 2018). Individual differences in personality, established by psychological instruments, can not only be indicators of the presence or absence of psychopathology, but can also indicate other significant aspects of an individual's condition, e.g., their feeling of satisfaction with their own life. If increased neuroticism can be connected with mental health problems, does a decrease in neuroticism lead to an increase of satisfaction with life? A model of impact of personality traits on satisfaction with life proposed by Costa and McCrae (1980) emphasizes the positive impact of extraversion and a negative impact of neuroticism on satisfaction with life. Bratko and Sabol (2006) confirmed in their research an association between extraversion, neuroticism and conscientiousness with life satisfaction. Campbell et al. (1976) according to Eddington and Shuman (2004) established that demographic factors (sex, age, education, race, income, marital status) explain <20% variance in the feeling of satisfaction with life. Furthermore, it was established that satisfaction with life has a relative stability over time although it can be moderately changed as a reaction to altered life circumstances (Pavot and Diener, 1993). Achieving positive subjective well-being includes positive experiences, like satisfaction with life and positive emotions (Diener, 1984). Personality traits, extraversion and neuroticism, impact affective experiences and a direction in the thinking process, and people rely on affective experiences and cognitive processes in their evaluation of satisfaction with their own life (Schimmack et al., 2002). One of the most common definitions of personality says that personality is a set of organized, proportionately permanent psychological traits and mechanisms within a person, which affects their interactions with the environment and adaptation to the environment (Larsen and Buss, 2005). Persistent patterns of behavior and experiencing of a person can be predicted from their personality traits. Hans Eysenck (1916–1997) claimed that certain personality models (for example, Cattell's model) contain too many similar factors and that a simple three-factor model can include all personality traits. Of all personality models, his had the strongest reliance on biology. He considered personality traits inheritable and having psycho-physiological basis. He stated three main personality features: extraversion/introversion (E), neuroticism/emotional stability (N), and psychoticism (P). It was the reliance of Eysenck's theory on biology that confirmed that the optimal level of arousal of an organism can be the cause of subjective experience of life satisfaction in young people engaged in recreational sports, even in difficult social conditions. It can be said that in this way they save themselves and show that there is hope for the humankind to remain in its original form.

## Conclusion

In conclusion, it can be said that the results are similar to the findings of other researchers which have established a significant predictive power of extraversion in the subjective experience of life satisfaction. Neuroticism and psychoticism are not significant predictors in predicting subjective feeling of life satisfaction in recreational athletes. The results represent a contribution to the understanding of the process of self-evaluation of satisfaction with life and can have a number of implications on different aspects of human life. They can also be a starting point in subsequent research of this construct. Furthermore, the results encourage people, regardless of demographic characteristics, to engage in physical activity in order to increase personal satisfaction with life.

## Data Availability Statement

The original contributions presented in the study are included in the article/supplementary material, further inquiries can be directed to the corresponding author.

## Ethics Statement

The studies involving human participants were reviewed and approved by Faculty of Sport and Physical Education, University of Niš, Serbia. The ethics committee waived the requirement of written informed consent for participation.

## Author Contributions

DŽ, JN, AĐ, BV, ŽB, and MB designed the research. DŽ, JN, and SP analyzed and interpreted the data, edited the manuscript, and approved the final version to be published and are accountable for all aspects of the work. DŽ, JN, BV, and MB performed the research and wrote the initial draft of the manuscript. All authors contributed to the article and approved the submitted version.

## Funding

The study was conducted within the project Anthropological characteristics of children in Sout heast Serbia - status, changes and trends (4-1206/21), funded and implemented by the Faculty of Sports and Physical Education of the University of Niš.

## Conflict of Interest

The authors declare that the research was conducted in the absence of any commercial or financial relationships that could be construed as a potential conflict of interest.

## Publisher's Note

All claims expressed in this article are solely those of the authors and do not necessarily represent those of their affiliated organizations, or those of the publisher, the editors and the reviewers. Any product that may be evaluated in this article, or claim that may be made by its manufacturer, is not guaranteed or endorsed by the publisher.

## References

[B1] Andrews F. M. Withey S. B. (1976). Social Indicators of Well-Being: Americans' Perceptions of Life Quality. New York, NY: PlenumPress.

[B2] Arbuckle J. L. (2014). Amos (version 23.0)[computer program]. Chicago: IBM SpSS.

[B3] Baudin N. Aluja A. Rolland J. P. Blanch A. (2011). The role of personality in satisfaction with life and sport. Psicol. Conduct. 19, 333–345.

[B4] Begčević I. (2021). Uloga ekstraverzije u objašnjavanju odnosa usamljenosti i zadovoljstva Životom (Unpublished master's thesis). University of Zagreb, Faculty of Humanities and Social Sciences, Departman of Psychology, Zagreb, Croatia.

[B5] Bender A. Beller S. (2011). The Cultural Constitution of Cognition: taking the Anthropological Perspective. Front. Psychol. 2:67. 10.3389/fpsyg.2011.00067 21716578 PMC3110796

[B6] Bratko D. Sabol J. (2006). Osobine ličnosti i osnovne psihološke potrebe kao prediktori zadovoljstva Životom: rezultati on-line istraŽivanja. Fam. Plan. Chassis Fut. Ministry 15, 693–711.

[B7] Campbell A. Converse P. E. Rodgers W. (1976). The Quality of American Life: Perceptions, Evaluations, and Satisfactions. New York, NY: Russell Sage Foundation.

[B8] Costa P. T. McCrae R. R. (1980). Influence of extraversion and neuroticism on subjective well-being: happy and unhappy people. J. Pers. Soc. Psychol. 38, 668–678. 10.1037/0022-3514.38.4.668 7381680

[B9] DeNeve K. M. Cooper H. (1998). The happy personality: a meta-analysis of 137 personality traits and subjective well-being. Psychol. Bull. 124, 197–229. 10.1037/0033-2909.124.2.197 9747186

[B10] Diener E. (1984). Subjective well-being. Psychol. Bull. 95, 542–575. 10.1037/0033-2909.95.3.542 6399758

[B11] Diener E. (2013). The remarkable changes in the science of subjective well-being. Perspect. Psychol. Sci. 8, 663–666. 10.1177/1745691613507583 26173230

[B12] Diener E. Diener C. (1996). Most people are happy. Psychol. Sci. 7, 181–185. 10.1111/j.1467-9280.1996.tb00354.x

[B13] Diener E. Emmons R. A. Larsen R. J. Griffin S. (1985). The satisfaction with life scale. J. Pers. Assess. 49, 71–75. 10.1207/s15327752jpa4901_13 16367493

[B14] Diener E. Larsen R. J. (1993). The experience of emotional well-being, in Handbook of Emotions, eds M. Lewis and J. M. Haviland (New York, NY: The Guilford Press), 405–415.

[B15] Diener E. Oishi S. Lucas R. E. (2003). Personality, culture, and subjective well-being: emotional and cognitive evaluations of life. Annu. Rev. Psychol. 54, 403–425. 10.1146/annurev.psych.54.101601.145056 12172000

[B16] Diener E. Ryan K. (2009). Subjective well-being: a general overview. S. Afr. J. Psychol. 39, 391–406. 10.1177/008124630903900402

[B17] Diener E. Suh E. M. Lucas R. E. Smith H. L. (1999). Subjective well-being: three decades of progress. Psychol. Bull. 125, 276–302. 10.1037/0033-2909.125.2.276

[B18] Dolan P. Kudrna L. Stone A. (2017). The measure matters: an investigation of evaluative and experience-based measures of wellbeing in time use data. Soc. Indic. Res. 134, 57–73. 10.1007/s11205-016-1429-8 28983145 PMC5599459

[B19] Downward P. Dawson P. (2016). Is it pleasure or health from leisure that we benefit from most? an analysis of well-being alternatives and implications for policy. Soc. Indic. Res. 126, 443–465. 10.1007/s11205-015-0887-8

[B20] Eddington N. Shuman R. (2004). Subjective Well-Being. Austin: Continuing Psychology Education.

[B21] Emić E. Selimović A. Berberović Dz. (2015). Reliability and Validity of Psychoticism, Extraversion, and Neuroticism (PEN) Scales in Eysenck's Personality Questionnaire (EPQ). Savremeni trendovi u psihologiji 2013—Current Trends in Psychology 2013. Novi Sad: Faculty of Philosophy Novi Sad, 267–269.

[B22] Emmons R. A. Diener E. (1985). Personality Correlates of Subjective Well-Being. Pers. Soc. Psychol. Bull. 11, 89–97. 10.1177/0146167285111008

[B23] Eysenck S. B. Eysenck H. J. Barrett P. (1885). A revised version of the Psychoticism Scale. Pers. and Individ. Dif. 6(1), 21–29.

[B24] Geron E. Furst D. Rotstein P. (1986). Personality of athletes participating in various sports. Int. J. Sport Psychol. 17, 120–135.

[B25] Gutiérrez-Cobo M. J. Megías-Robles A. Gómez-Leal R. Cabello R. Fernández-Berrocal P. (2021). Is it possible to be happy during the COVID-19 lockdown? a longitudinal study of the role of emotional regulation strategies and pleasant activities in happiness. Int. J. Environ. Res. Public Health 18:3211. 10.3390/ijerph18063211 33808852 PMC8003758

[B26] Hall C. S. Lindzey G. (1978). Theories of Personality. New York, NY: John Willey and Sons.

[B27] Headey B. Muffels R. Wagner G. G. (2013). Choices which change life satisfaction: similar results for Australia, Britain and Germany. Soc. Indic. Res. 112, 725–748. 10.1007/s11205-012-0079-8

[B28] Headey B. W. (2006a). Subjective well-being: revisions to dynamic equilibrium theory using national panel data and panel regression methods. Soc. Indic. Res. 79, 369–403. 10.1007/s11205-005-5381-2

[B29] Headey B. W. (2006b). Happiness: Revising Set Point Theory and Dynamic Equilibrium Theory to Account for Long Term Change. DIW Discussion Paper No. 607. Berlin: DIW.

[B30] Headey B. W. (2008). Life goals matter to happiness: a revision of set-point theory. Soc. Indic. Res. 86, 213–231. 10.1007/s11205-007-9138-y

[B31] Höner O. Demetriou Y. (2012). Körperlich-sportliche Aktivität und gesundheitsbezogene Lebensqualität, in Seelische Gesundheit und sportliche Aktivität, eds R. Fuchs and W. Schlicht (Göttingen: Hogrefe), 34–55.

[B32] Huang H. Humphreys B. R. (2011). Sport participation and happiness: evidence from US micro data, in The Economics of Sport, Health, and Happiness: The Promotion of Well-Being Through Sporting Activities, eds P. Rodriguez, S. Kesenne, and B. R. Humphreys BR (Glos: Edward Elgar Publishing), 163–183.

[B33] Jetzke M. Mutz M. (2020). Sport for pleasure, fitness, medals or slenderness? differential effects of sports activities on well-being. Appl. Res. Qual. Life 15, 1519–1534. 10.1007/s11482-019-09753-w

[B34] Jovanović V. (2016). The validity of the Satisfaction with Life Scale in adolescents and a comparision with single-item life satisfaction measures: a preliminary stady. Qual. Life Res. 25, 3173–3180. 10.1007/s11136-016-1331-5 27262574

[B35] Larsen R. J. Buss D. M. (2005). Personality Psychology: Domains of Knowledge About Human Nature, 2nd Edn. New York, NY: McGraw Hill.

[B36] Lep Ž. Babnik K. Hacin Beyazoglu K. (2020). Emotional responses and self-protective behavior within days of the COVID-19 outbreak: the promoting role of information credibility. Front. Psychol. 11:1846. 10.3389/fpsyg.2020.01846 32849087 PMC7411328

[B37] Lera-Lopez F. Ollo-López A. Garrués-Irisarri M. Cabasés J. M. Sánchez E. (2019). How the relationship between physical activity and health changes with age. Eur. J. Ageing 16, 3–15. 10.1007/s10433-018-0471-6 30886556 PMC6397114

[B38] Lodha P. Karia S. (2019). Testosterone and schizophrenia: A clinical review. Annals of Indian Psychiatry 3:92.

[B39] Marques A. Martins J. Peralta M. Catunda R. Nunes L. S. (2016). European adults' physical activity sociodemographic correlates: a cross-sectional study from the European Social Survey. PeerJ 4:e2066. 10.7717/peerj.2066 27280072 PMC4893333

[B40] Mascia L. M. Agus M. Penna M. P. (2020). Emotional intelligence, self-regulation, smartphone addiction: which relationship with student well-being and quality of life? Front. Psychol. 11:375. 10.3389/fpsyg.2020.00375 32210888 PMC7068808

[B41] McMahon E. M. Corcoran P. O'Regan G. Keeley H. Cannon M. Carli V.. (2017). Physical activity in European adolescents and associations with anxiety, depression and well-being. Eur. Child Adolesc. Psychiatry 26, 111–122. 10.1007/s00787-016-0875-9 27277894

[B42] Milas G. (2009). IstraŽivačke metode u psihologiji i drugim društvenim znanostima. [Research methods in psychology and other social sciences]. Jastrebarsko: Naklada Slap.

[B43] Morgan G. (1980). Paradigms, metaphors, and puzzle solving in organization theory. Adm. Sci. Q. 605–622. 10.2307/2392283

[B44] Nederlof A. F. Muris P. Hovens J. E. (2011). Threat/control-override symptoms and emotional reactions to positive symptoms as correlates of aggressive behavior in psychotic patients. J. Nerv. Ment. Dis. 199, 342–347. 10.1097/NMD.0b013e3182175167 21543954

[B45] Oishi S. Diener E. Lucas R. E. (2007). The optimum level of well-being: can people be too happy? Perspect. Psychol. Sci. 2, 346–360. 10.1111/j.1745-6916.2007.00048.x 26151972

[B46] Pavot W. Diener E. (1993). Review of the satisfaction with life scale. Psychol. Assess. 2, 164–172. 10.1037/1040-3590.5.2.164

[B47] Pavot W. Diener E. (2013). Happiness experienced: the science of subjective well-being, in The Oxford Handbook of Happiness, eds S. David, I. Boniwell, and A. C. Ayers (Oxford: Oxford University Press), 134–151.

[B48] Rice S. M. Purcell R. De Silva S. Mawren D. Mc. Gorry P. D. Parker A. G. (2016). The mental health of elite athletes: a narrative systematic rewiew. Sports Med. 46, 1333–1353. 10.1007/s40279-016-0492-2 26896951 PMC4996886

[B49] Richards J. Jiang X. Kelly P. Chau J. Bauman A. Ding D. (2015). Don't worry, be happy: cross-sectional associations between physical activity and happiness in 15 European countries. BMC Public Health 15:53. 10.1186/s12889-015-1391-4 25636787 PMC4320474

[B50] Sato M. Jordan J. S. Funk D. C. Sachs M. L. (2018). Running involvement and life satisfaction: the role of personality. J. Leis. Res. 49, 28–45. 10.1080/00222216.2018.1425051

[B51] Schimmack U. Diener E. Oishi S. (2002). Life-satisfaction is a momentary judgment and a stable personality characteristic: the use of chronically accessible and stable sources. J. Pers. 70, 345–384. 10.1111/1467-6494.05008 12049164

[B52] Schimmack U. Oishi S. Furr R. M. Funder D. C. (2004). Personality and life satisfaction: a facet-level analysis. Pers. Soc. Psychol. Bull. 30, 1062–1075. 10.1177/0146167204264292 15257789

[B53] Servaas M. N. Van Der Velde J. Costafreda S. G. Horton P. Ormel J. Riese H.. (2013). Neuroticism and the brain: a quantitative meta-analysis of neuroimaging studies investigating emotion processing. Neurosci. Biobehav. Rev. 37, 1518–1529. 10.1016/j.neubiorev.2013.05.005 23685122

[B54] Sigvartsen J. Gabrielsen L. E. Abildsnes E. Stea T. H. Omfjord C. S. Rohde G. (2016). Exploring the relationship between physical activity, life goals and health-related quality of life among high school students: a cross-sectional study. BMC Public Health 16:709. 10.1186/s12889-016-3407-0 27488255 PMC4972944

[B55] Šipka P. (1985). Prevod i adaptacija Eysenckovog testa ličnosti EPQ-R. Beograd: Vojnomedicinska akademija.

[B56] Sit C. H. Lindner K. J. (2005). Motivational orientations in youth sport participation: using achievement goal theory and reversal theory. Pers. Individ. Dif. 38, 605–618. 10.1016/j.paid.2004.05.015

[B57] Steel P. Schmidt J. Shultz J. (2008). Refining the relationship between personality and subjective well-being. Psychol. Bull. 134, 138–161. 10.1037/0033-2909.134.1.138 18193998

[B58] Tajima-Pozo K. Bayón C. Díaz-Marsá M. Carrasco J. L. (2015). Correlation between personality traits and testosterone concentrations in healthy population. Indian J. Psychol. Med. 37, 317–321. 10.4103/0253-7176.162956 26664080 PMC4649825

[B59] Tuce D. Fako I. (2014). Odrednice zadovoljstva Životom kod adolescenata, [Determinants of life satisfaction in adolescents]. Psihol. Teme 23,407–493.

[B60] Vinson T. Ericson M. (2014). The social dimensions of happiness and life satisfaction of Australians: evidence rom the World Values Survey. Int. J. Soc. Welf. 23, 240–253. 10.1111/ijsw.12062

[B61] White R. L. Babic M. J. Parker P. D. Lubans D. R. Astell-Burt T. Lonsdale C. (2017). Domain-specific physical activity and mental health: a meta-analysis. Am. J. Prev. Med. 52, 653–666. 10.1016/j.amepre.2016.12.008 28153647

[B62] Wiese C. W. Kuykendall L. Tay L. (2018). Get active? a meta-analysis of leisure-time physical activity and subjective well-being. J Posit. Psychol. 13, 57–66. 10.1080/17439760.2017.1374436

[B63] Zhang Z. Chen W. (2019). A systematic review of the relationship between physical activity and happiness. J. Happiness Stud. 20, 1305–1313. 10.1007/s10902-018-9976-0

